# How Calcifications Guide the Diagnosis: A Case of Gorlin's Cyst

**DOI:** 10.1002/ccr3.70369

**Published:** 2025-04-22

**Authors:** Rym Kammoun, Manel Gharbi, Rawia Jaziri, Nawress Ghadhab, Imen Chaabani, Touhami Ben Alaya

**Affiliations:** ^1^ Laboratory of Histology and Embryology Faculty of Dental Medicine of Monastir, University of Monastir Monastir Tunisia; ^2^ ABCDF Laboratory for Biological Clinical and Dento‐Facial Approach University of Monastir Monastir Tunisia; ^3^ Department of Radiology University Dental Clinic Monastir Tunisia; ^4^ Unity of Bioactive Natural Substances and Biotechnology Faculty of Dental Medicine, University of Monastir Monastir Tunisia

**Keywords:** odontogenic cyst calcifying, osteolysis, radiography panoramic, tomography X‐ray computed

## Abstract

Calcified odontogenic epithelial cyst known as Gorlin's cyst is one of the benign odontogenic tumors of the maxillae. In imaging, the most revealing aspect is a well‐limited osteolytic image with peripheral calcifications. The aim of the study was to highlight these radiological features to establish the correct diagnosis and appropriate treatment.

**Summary**

- In the case of a well‐limited mixed image showing calcifications in the periphery of the lesion with fluid content on sectional imaging (CT scan), the essential diagnosis to be made by the practitioner is that of a Gorlin Cyst.

A 30‐year‐old patient in good general condition presented with a right maxillary deformity that had been progressing without symptoms over 1 year.

Exobuccal examination revealed a right genital swelling hard to palpation. Endobuccal examination revealed a right maxillary vestibule filling with local depressivity of the vestibular and lingual tables (ping pong ball sensation).

The panoramic view showed a well‐limited osteolytic image in some areas, extending from the molar to the incisor region, with the presence of calcifications of a bony nature (Figure 1A).

**FIGURE 1:**
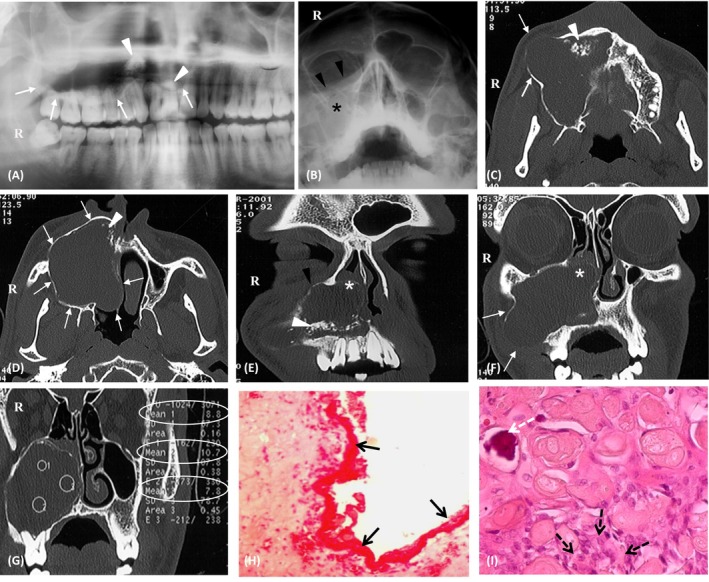
(A) Panoramic radiography, lower limit of the right maxillary osteolytic image (white arrows) with the presence of intra‐lesional calcifications (white arrowheads). (B) Blondeau incidence, total filling of the right maxillary sinus (black star) with upward displacement of the orbital floor (black arrowheads). (C, D) Axial CT sections, osteolytic image with displacement and thinning of the outer cortex without rupture (white arrows), presence of peripheral calcifications (white arrowhead). (E, F) Coronal CT sections, osteolysis affecting the nasal cavities (white star) and displacement of the orbital floor (black arrowhead). (G) Coronal CT section, three levels of density measurement within the lesion (circles 1, 2, 3), Density 1: 808 HU, Density 2: 10.7 HU, and Density 3: 708 HU. (H) Microscopic observation at low magnification (HE × 100) showing Cystic wall lined with epithelium of variable thickness. (black arrows). (I) At high magnification (HE × 400): Groups of ghost cells, Some of the ghost underwent calcification (dashed white arrows), With multinucleated giant cell reaction (dashed black arrows).

The clinical and radiological findings point to the diagnosis of a slowly progressive, non‐aggressive lesion, probably containing fluid. The presence of intra‐lesional calcifications suggests two probable diagnoses: adenomatoid odontogenic tumor or Gorlin's cyst.

A CT scan revealed a well‐limited osteolytic image occupying the entire right maxilla (Figure 1C) and extending into the homolateral nasal cavity, with compression and thinning of the external cortex (Figure 1D–F) and upward displacement of the orbital floor (Figure 1B,E,F). The CT also showed intra‐lesional and peripheral calcifications (Figure 1C–E). Density measurements concluded that the lesion was fluid in nature (Figure 1G). Biopsy confirmed the diagnosis of Gorlin's cyst (Figure 1H,I).

Gorlin cyst, which is also recognized by the terms calcifying epithelial odontogenic cyst and calcifying ghost cell odontogenic cyst, is a rare developmental lesion that arises from the odontogenic epithelium. It occurs with an equal incidence in the maxilla and the mandible. Approximately most cases are discovered in the incisor and canine areas of the jaws and usually arise intraosseously; however, it may occur extraosseously, too. It has a peak incidence during the second and third decades of life. It is clinically characterized as a painless, slow‐growing cystic lesion. Radiographically, calcification is an important radiographic feature for the interpretation of Gorlin's cyst [1]. Analysis of the lesional content allows for a better diagnostic orientation, essentially through the possibility of density measurement provided by the scanner. In fact, the presence of a liquid‐dense lesion content and calcifications of a bony nature located at the periphery of the lesion because—they are encrusted in the epithelial wall points—to the diagnosis of a Gorlin cyst, whereas a lesion with heterogeneous firm content containing central calcifications dispersed throughout the image is more suggestive of a solid, non‐cystic pathology [2].

In the present case, the lesion was non‐aggressive, slowly progressive, and asymptomatic, leading to a delay in consultation and a consequent increase in the size of the image. CT scan is therefore essential for identifying the specific characteristics of the lesion and its extension, allowing accurate diagnosis and appropriate treatment.

## Author Contributions

**Rym Kammoun:** conceptualization, methodology, writing – original draft. **Manel Gharbi:** formal analysis. **Rawia Jaziri:** formal analysis. **Nawress Ghadhab:** formal analysis. **Imen Chaabani:** methodology, validation, visualization. **Touhami Ben Alaya:** methodology, supervision, validation, visualization.

## Consent

A written informed consent was obtained in accordance with the journal's patient consent policy.

## Conflicts of Interest

The authors declare no conflicts of interest.

## Data Availability

Data available on request from the authors; the data that support the findings of this study are available from the corresponding author upon reasonable request.
